# Long-Term Mating Orientation in Men: The Role of Socioeconomic Status, Protection Skills, and Parenthood Disposition

**DOI:** 10.3389/fpsyg.2022.815819

**Published:** 2022-02-25

**Authors:** Gabriela Fajardo, Pablo Polo, José Antonio Muñoz-Reyes, Carlos Rodríguez-Sickert

**Affiliations:** ^1^Laboratorio de Comportamiento Animal y Humano, Facultad de Gobierno, Centro de Investigación en Complejidad Social, Universidad del Desarrollo, Santiago, Chile; ^2^Laboratorio de Comportamiento Animal y Humano, Centro de Estudios Avanzados, Universidad de Playa Ancha, Viña del Mar, Chile

**Keywords:** strategic pluralism, sociosexuality, mating strategies, socioeconomic status, strength

## Abstract

From an evolutionary perspective, phenotypic, social, and environmental factors help to shape the different costs and benefits of pursuing different reproductive strategies (or a mixture of them) from one individual to another. Since men’s reproductive success is mainly constrained to women’s availability, their mating orientations should be partially calibrated by features that women prefer in a potential partner. For long-term relationships, women prefer traits that signal access to resources, protection skills, and the willingness to share them. Using generalized linear models with laboratory data taken from a Chilean population (*N* = 197), this study aimed to test whether real and potential resources (measured as self-reported socioeconomic status), protection skills (measured as handgrip strength), and the willingness to provide resources and protection (measured as their disposition toward parenthood) are related to mating orientation in men. Our predictions were: (1) socioeconomic status would be positively associated with long-term and short-term mating orientation but for long-term-oriented individuals, this would be enhanced by having a more favorable parenthood disposition and (2) strength would be positively related to long-term mating orientation in men with higher socioeconomic status and a favorable disposition toward parenthood and it would have a positive and direct association with short-term mating orientation. Our results partially supported the first hypothesis, since men with higher socioeconomic status were more long-term oriented, but parenting disposition did not moderate this effect. Contrary to our expectations, socioeconomic status was not related to short-term mating orientation. Strength appeared not to be significant for long-term mating orientation, even interacting with other traits. However, strength by itself was powerfully linked with a short-term mating orientation. Our results suggest that only some individuals that are attractive for long-term relationships are indeed long-term oriented and may reflect the overall conflict of interests between mating strategies among sexes.

## Introduction

In sexually reproducing species, individuals can allocate time and energy toward different reproductive activities such as finding and selecting mates, maintaining a pair-bonded relationship, or investing in parental care. Since time and energy are limited, this creates trade-offs between different aspects of reproduction (Stearns, 1992). One important reproductive trade-off is between mating and parenting. In this regard, humans show a wide variety of mating strategies, from the establishment and maintenance of long-lasting pair-bonds (long-term strategies)—with different degrees of parental investment from men and women—to promiscuous mating (short-term strategies; Buss and Schmitt, 1993; Schmitt, 2015) that can be measured through differences in sociosexuality (individual differences in the willingness to engage in uncommitted sex; Simpson and Gangestad, 1991). This trade-off was shaped differently between the sexes since they faced divergent adaptative problems (Buss and Schmitt, 2019). In this regard, men are, on average, more prone to display short-term mating strategies than women mainly due to differences in obligated parental investment (Trivers, 1972; Clutton-Brock and Vincent, 1991). However, usually, within-sex individual differences are greater in magnitude than between-sex differences suggesting that mating strategies in humans are expressed in a highly variable and flexible fashion (Schmitt, 2015). From an evolutionary perspective and following the strategic pluralism hypothesis (SPH), this variation can be partially explained by phenotypic, social, and environmental factors, including physical features, social status, and mating opportunities that shape different costs and benefits in terms of pursuing different reproductive strategies—or a mixture of them—from one individual to another (Gangestad and Simpson, 2000).

As men’s reproductive success depends mainly on access to partners, their mating orientations should be partially calibrated by features that women prefer in them (Buss and Schmitt, 2019). These preferences vary depending on the context, with different traits being preferred for short and long-term relationships. For short-term mating, women prefer characteristics associated with “good genes” and physical condition, such as low facial asymmetry—related to developmental stability and diseases (Van Dongen and Gangestad, 2011; but see for opposite or mixed results; Pound et al., 2014; Foo et al., 2017)—, and muscularity or strength—associated with high levels of pubertal testosterone (Evans, 2004). Traits related to good genes are also preferred for long-term relationships but they are less important than for short-term ones and other features become more relevant, such as economic resources, physical protection (also linked to good genes traits), and willingness to allocate those resources to a single partner and their offspring (Buss and Schmitt, 2019). Whereas several studies found evidence that features preferred by women for short-term mating are, in turn, related to men’s short-term mating orientation (Hughes and Gallup, 2003; Frederick and Haselton, 2007; Lukaszewski et al., 2014; Polo et al., 2019), the relationship between traits preferred by women for long-term mating and men’s mating orientation are less empirically explored. In this study, we focused on pinpointing if traits attractive for women for long-term relationships are related to men’s long-term mating orientation in order to shed light on the factors that may calibrate the expression of long-term mating strategies in men.

First, economic resources are more relevant to women than to men when it comes to long-term partner selection, compared to short-term mating contexts (Townsend, 1989; Buss et al., 1990; Sprecher et al., 1994; Buunk et al., 2002; Li and Kenrick, 2006). This pattern of sex-differences has been found in numerous cultures with different degrees of gender inequalities, different mating systems, and religions (Buss, 1989; Wang et al., 2018). Resources are generally measured as present income or other material assets, and numerous studies point toward the importance of having resources to be considered a good long-term partner to women (Kenrick et al., 1990; Townsend and Levy, 1990; Li et al., 2002; Hitsch et al., 2010; Anderson and Klofstad, 2012; Fales et al., 2016). Furthermore, economic resources may influence women’s perception of men’s attractiveness (Dunn and Searle, 2010; Shuler and McCord, 2010; Dunn and Hill, 2014; Wang et al., 2018). Other measures of economic resources are traits such as the thriving or ability to generate resources—measured as educational level, intelligence, ambition, or industriousness—and are deemed similarly desirable by women while choosing a long-term partner (Marlowe, 2004; Souza et al., 2016). In addition to this evidence that links resources with long-term preferences, other studies suggest that economic resources may also be preferred by women in short-term partners. In this regard, Greiling and Buss (2000) found empirical evidence that one of the possible functions of short-term mating for women is to obtain resources from casual partners (resource acquisition hypothesis) and, therefore, men signaling resources or status may be preferred for short-term relationships as well. Another study found that priming women with wealth cues produced a shift toward selecting more mates for short-term relationships (Thomas and Stewart-Williams, 2018). In addition, having resources may alleviate the cost of pursuing a short-term mating strategy in men. All these pieces of evidence combined suggest that economic resources may be important for the expression of both long-term and short-term mating strategies in men. To our knowledge, only three studies have reported a link between resources and men’s mating strategies measured through their sociosexuality. In one of these studies, Townsend (1993) found that, among college students, those that reported higher expected income had more unrestricted sociosexuality suggesting a preference for short-term mating. However, the father’s education and income were not related to sociosexuality. In more recent studies, Sprecher et al. (2013) found a null effect of socioeconomic status on sociosexuality, and Szepsenwol et al. (2017) found that early and current environmental predictability but not early socioeconomic status predicted restricted sociosexuality during adulthood; however, they did not consider current socioeconomic status in their analyses. Moreover, neither of these studies specifically aimed to test the relationship between current socioeconomic status and sociosexuality and they used a unidimensional measure of sociosexuality precluding an assessment of whether economic resources are important for both short and long-term mating. More indirect evidence about the role of economic resources in sociosexuality comes from studies using the Wealth subscale included in the Components of Mate Value Survey (Fisher et al., 2008). Fisher et al. (2008) found a positive relationship between this subscale and the number of long-term relationships reported by women but not by men. In addition, a recent meta-analysis found that mate value was related to unrestricted sociosexuality (Arnocky et al., 2021), but is not clear if the Wealth component of mate value contributes to this relationship.

Another relevant feature preferred in a long-term mate is being able to provide physical protection to a single partner and their offspring against external threats, giving them an advantage for survival (Buss and Schmitt, 2019). Men’s ability to provide this protection is mainly related to the strength of the upper-body (Puts, 2010; Sell et al., 2012), which is highly correlated with handgrip strength (Gallup and Fink, 2018). Since protection is always given against external threats—including, but not exclusively, other men—, protection traits are also related to higher intrasexual competitive abilities (Muñoz-Reyes et al., 2019). As mentioned before, previous studies usually link strength and muscularity with women’s preferences for short-term mating and with attitudes and behavior related to short-term mating strategies in men (Hughes and Gallup, 2003; Frederick and Haselton, 2007). Most of these studies did not consider short and long-term mating orientation as two separate—and sometimes simultaneous—dimensions of sociosexuality. However, the only two which took that approach did not find a link between strength or muscularity with long-term sociosexual orientation (Lukaszewski et al., 2014; Polo et al., 2019). This suggests that, if physical traits denoting fighting and protection skills are playing a role in long-term mating strategies, their effect may be contingent upon the possession of other relevant traits in this context such as resources. In other words, protection skills could be relevant for the expression of long-term mating especially in those men that also have real or potential resources.

As important as resources and protection-related traits themselves are for women, there are personality features that signal a willingness to allocate those resources and abilities in one partner and their offspring over a significant period (Buss, 2018; Webb and Fisher, 2018; Zinck et al., 2021). There is cross-cultural evidence that relevant personality traits which women prefer when choosing a long-term partner are kindness, understanding, and commitment (Buss et al., 1990; Kenrick et al., 1990; Simpson and Gangestad, 1992; Schmitt, 2005; Buss and Schmitt, 2019). In this regard, a positive parenthood disposition denotes the willingness to invest in offspring and it is considered an especially relevant component of men’s mate value to attract partners for long-term relationships (Fisher et al., 2008). Accordingly, parenthood disposition may be crucial in moderating the effects of resources and protection-related traits in the expression of long-term mating orientation in men.

To sum up, women’s preferences in men for long-term relationships are mainly related to economic resources, protection, and the willingness to share those resources and protection with offspring. Accordingly, and following the proposal of SPH, men showing these traits should be more oriented toward long-term mating, as these traits are preferred by women in this context. Moreover, considering a multidimensional measure of sociosexuality, traits denoting resources and protection skills are also expected to be related to short-term mating. However, there is little evidence of whether these characteristics are associated with men’s long-term mating orientation as a separate dimension from short-term mating orientation. This study aims to test if resources—measured as self-reported socioeconomic status—, protection skills—signaled by handgrip strength—and the willingness to share resources and protection—measured indirectly through a parenthood disposition measure from the Components of Mate Value Survey (Fisher et al., 2008)—are related to long-term mating orientation in men, using generalized linear models with laboratory data taken from a Chilean population. Following our argument that the willingness to allocate resources in offspring is a key trait denoting commitment, our first specific prediction was that self-reported socioeconomic status would be positively associated both with long and short-term sociosexual orientation, but for long-term-oriented individuals this would be enhanced by having a more favorable parenthood disposition. As our second prediction, we expected that handgrip strength would be positively related to long-term mating orientation in those men with high self-reported socioeconomic status with a positive parenthood disposition moderating this effect, but we expected that handgrip strength would be directly and positively linked to short-term mating orientation.

## Materials and Methods

### Participants

The complete dataset was composed of 212 men between 18 and 38 years old (*M* = 22.52; *SD* = 4.65). Given the purpose of this study, we selected heterosexual and bisexual men (*N* = 197). The data was collected in 2016 in Chile’s Valparaiso region through an open call in universities and public places, so we had a wider, more diverse sample from a general population. All the procedures were performed in the laboratory at the institution. Participants signed informed consent forms and the study protocol was approved by the Universidad de Playa Ancha Ethics Committee.

First, participants answered a sociodemographic questionnaire that included information about age, gender, sexual orientation, and relationship status (56.8% single). Then, they answered psychometric questionnaires followed by anthropometric measures. At the end of the session, they received a 5,000 Chilean peso show-up fee (around $6.10 USD).

### Measures

#### Sociosexual Orientation

The relative investment in mating vs. parenting can be measured through the individual expression of sociosexuality. We used Jackson and Kirkpatrick’s (2007) Multidimensional Sociosexual Orientation Inventory (MSOI), which fulfills the need for a more complex and multidimensional construct to measure sociosexual attitudes and behavior. It consists of a questionnaire made up of 22 items that can be separated into three dimensions: short-term mating orientation (10 items), long-term mating orientation (7 items), and sociosexual behavior (5 items). The first two dimensions were attitudinal and consisted of a 7-point Likert scale (1 = strongly disagree to 7 = strongly agree) with statements like “I could easily imagine myself enjoying one night of sex with someone I would never see again,” for the short-term mating orientation dimension, or “I can see myself settling down romantically with one special person,” for the long-term mating orientation dimension. The sociosexual behavior dimension consisted of open numeric questions. Internal consistency, measured with Cronbach’s alpha, was high for both short-term (α = 0.90) and long-term (α = 0.84) mating orientation, and comparable with that provided in the original questionnaire (short-term: α = 0.94; long-term: α = 0.88).

#### Socioeconomic Status

As our measurement of real and potential resources, we employed the MacArthur Scale of Subjective Social Status (Adler and Stewart, 2007; Giatti et al., 2012), adapted to the Chilean population. This self-reported measurement consists of a ladder with 10 steps representing the place that participants locate themselves in society and their local social environment in terms of job, income, and educational level. The top of the ladder represents the people who have more money, more education, and better jobs while the bottom represents the people with less money, less education, and worse jobs or who are unemployed. We considered responses regarding the participant’s social position in overall society. We expected to have greater heterogeneity in socioeconomic status at the general level than at the local level due to the high socioeconomic inequalities in Chile (PNUD, 2018). In any case, results taking into account local socioeconomic status instead of global socioeconomic status do not differ in the relationships found.

#### Parenthood Disposition

To capture the willingness to invest in offspring, we used the Parenting subscale from the Components of Mate Value Survey (Fisher et al., 2008). In this subscale, participants responded on a 7-point Likert scale (1 = strongly disagree to 7 = strongly agree) to three statements. We selected two for this study: “I would make a good parent” and “It is important that the opposite sex views me as a good parent.” We excluded the parenting statement “I want to have children in my lifetime” since, as Fisher et al. (2008) mention, it is possible that a person who wants to have children might not necessarily take care of them and we were more interested in the individual valuation of parenting. The Pearson correlation coefficient between the two considered items was 0.43, that represents a moderate association (Dancey and Reidy, 2007). It is worth mentioning that neither of these parenting questions is included in the MSOI items.

#### Protection Skills

We measured participants’ handgrip strength (in kilograms) with a hydraulic handgrip dynamometer (Jamar 5030J1) following the procedure of Gallup et al. (2007). Individuals were instructed to be in a stand-up position and to squeeze the dynamometer with their forearm at a 90° angle to the body. We registered three measurements for each hand, with a 1-min rest between each strength test. We used the highest handgrip strength score.

#### Body Mass Index

This is a trait that covaries with strength and, therefore, it is convenient to control its effect when studying strength-relationships with other traits (Lassek and Gaulin, 2009). The participants’ weight (in kilograms) was measured using a digital body scale and their height (in centimeters) was obtained using a stadiometer (SECA 213).

### Data Analysis

First, we reported Pearson correlation coefficients between the continuous variables employed in this study and *t*-test for independent samples to describe differences in these variables according to the relationship status of individuals. Second, to test our hypotheses, we did a between-subjects analysis using generalized linear models with long and short-term mating orientation as dependent variables. Regarding the first hypothesis, we considered global socioeconomic status as an independent variable, and age (in years) and relationship status as control variables. For both dependent variables, we first tested the main effect of socioeconomic status, and then for long-term mating orientation, we included the expected interaction between self-reported status and parenthood disposition to test the moderation effect of parenthood disposition. To test our second hypothesis, we considered strength as an independent variable and Body Mass Index (BMI), age, and relationship status as control variables. We first tested the main effect of strength, and then, for long-term mating orientation, we included the expected interaction between strength, parenthood disposition, and self-reported socioeconomic status to test the moderator effects of parenthood disposition and self-reported socioeconomic status. Relationship status and age were statistically controlled because previous studies showed that they may have an effect over different aspects of sociosexuality (e.g., Simpson and Gangestad, 1991; Penke and Asendorpf, 2008; Polo et al., 2019).

We used generalized linear models because the responses of the dependent variables were independent (i.e., every case was independent), it does not need to satisfy homogeneity of variance nor normality of errors, and it uses maximum likelihood estimation (MLE) instead of ordinary least squares (OLS) to estimate the parameters. We reported standardized regression coefficients for all the models. We used the Pearson Correlation test to test collinearity between independent variables and the results were non-significant, with every coefficient under 0.30.

Finally, given the results obtained, we carried out an additional exploratory analysis to investigate the possible mediating role of parenthood disposition in the relationship between self-reported socioeconomic status and long-term mating orientation. Mediation analysis was performed using linear regressions and a bootstrapping method (5,000 bootstraps and *p* = 95%) to estimate the significance of the indirect effect.

Mediation analysis was performed with PROCESS macro (version 3.5) for SPSS (Hayes, 2017). All the remaining analyses were performed with R version 3.5.2 employing standard libraries and sjPlot package (Lüdecke, 2021), considering two-tailed tests with a level of significance set up at α < 0.05.

## Results

A descriptive summary of the variables employed in this study and their mean differences according to the relationship status of the individuals are shown in Table 1. Associations between continuous variables are shown in Table 2.

**TABLE 1:** Mean, standard deviation, and mean differences between paired (*N* = 112) and single (*N* = 85) individuals for each variable.

	Mean (*SD*)	Mean differences (single–paired)
Long-term mating orientation	5.61 (1.42)	0.58**
Short-term mating orientation	4.44 (1.42)	–0.01
Parenthood disposition	10.87 (2.79)	0.17
Global socioeconomic status	5.8 (1.65)	0.37
Handgrip Strength (in kg)	42.8 (7.45)	–0.52
BMI	24.2 (3.51)	–0.45
Age (in years)	22.5 (4.5)	−2.14**

*BMI, Body mass index.*

**p < 0.01.

**TABLE 2:** Pearson’s correlation coefficients between variables (*N* = 197).

	LTMO	STMO	PD	Global status	Strength	BMI	Age
LTMO		0.04	0.40***	0.23**	–0.05	–0.03	<0.01
STMO			0.10	0.07	0.13	–0.01	0.04
PD				0.28***	0.02	0.08	–0.03
Global status					0.11	–0.00	–0.04
Strength						0.17*	0.04
BMI							0.42***

*LTMO, Long term mating orientation; STMO, Short-term mating orientation; PD, Parenthood disposition; BMI, Body mass index.*

*p < 0.05, **p < 0.01, ***p < 0.001.

### Relationship Between Resources and Mating Orientations

Table 3 shows the main and interaction effects of socioeconomic status for long and short-term mating orientations. Regarding the main effects, we found a significant effect of socioeconomic status (β = 0.33, *t* = 3.24, *p* = 0.001) on the expression of long-term mating orientation. According to our prediction, individuals with higher socioeconomic status reported higher levels of long-term mating orientation. Regarding short-term mating orientation, we found no main effect on it in terms of socioeconomic status (β = 0.08, *t* = 0.76, *p* = 0.45).

**TABLE 3:** Generalized linear models, standardized coefficients and standard errors for long and short-term sociosexual orientation (*N* = 197).

	First hypothesis: testing socioeconomic status		

		Long-term orientation	Short-term orientation
Main effects	Global status	0.33** (0.10)	0.08 (0.10)
	Age	0.01 (0.10)	0.13 (0.10)
	In a relationship	0.04 (0.10)	−0.31** (0.10)
	Intercept	5.61*** (0.10)	4.44*** (0.10)
	AIC	696.1	695.6
Interaction effects	Global status * Parenthood disposition	−0.03 (0.09)	−
	Intercept	5.62*** (0.10)	−
	AIC	673.1	−

	**Second hypothesis: testing protection skills (strength)**		

		**Long-term orientation**	**Short-term orientation**

Main effects^a^	Strength	−0.06 (0.10)	0.21* (0.10)
	BMI	−0.05 (0.11)	−0.09 (0.11)
	Intercept	5.61*** (0.10)	4.44*** (0.10)
	AIC	707.9	693.6
Interaction effects^b^	Global status * Strength	−0.20^∼^ (0.11)	−
	Parenthood disposition * Strength	0.09 (0.11)	
	Global status * Parenthood disposition* Strength	−0.02 (0.10)	−
	Intercept	5.65*** (0.10)	−
	AIC	677.1	−

Standard errors in parenthesis. ***p < 0.001; **p < 0.01; *p < 0.05; ^∼^p < 0.10.

*^a^Models with strength and BMI also included control variables (relationship status and age).*

*^b^For more details (p-values, control variables and two-way interactions) (see the full table in Supplementary Table 1).*

When considering the predicted interaction for long-term mating orientation, the results showed that parenthood disposition did not moderate the effect of socioeconomic status on long-term mating orientation (β = −0.03, *t* = −0.29, *p* = 0.775).

The exploratory mediation analysis showed that the effect of socioeconomic status on long-term mating orientation was mediated by parenthood disposition (indirect effect; β = 0.10, Bootstrapped *SE* = 0.03, 95% bootstrapped CI [0.05, 0.17]). On the one hand, socioeconomic status was positively related to parenthood disposition (β = 0.28, *t* = 3.95, *p* < 0.001), but its direct effect on long-term mating orientation only reached a statistical trend (β = 0.13, *t* = 1.88, *p* = 0.061). On the other hand, parenthood disposition was related to long-term mating orientation (β = 0.36, *t* = 5.30, *p* < 0.001).

### Relationship Between Protection Skills and Mating Orientation

Table 3 shows the main and interaction effects of strength over short and long-term mating orientation. First, we found that there was no main effect of strength on long-term mating orientation (β = −0.06, *t* = −0.10, *p* = 0.569), but it indeed had a significant main effect on short-term mating orientation, showing that stronger men had a higher short-term mating orientation (β = 0.21, *t* = 2.06, *p* = 0.041). When including the predicted three-way interaction between strength, socioeconomic status and parenthood disposition with long-term mating orientation, we found a non-significant effect (β = −0.02, *t* = −0.24, *p* = 0.811). We also found no significant effects in any of the two-way interactions.

## Discussion

The SPH proposes that the relative allocation of time and energy between mating and parenting activities is facultatively calibrated by an individual’s particular traits and social context. In this regard, men showing traits preferred by women for long-term relationships should display higher sociosexual attitudes toward long-term mating (Gangestad and Simpson, 2000; Buss and Schmitt, 2019). Previous studies reporting associations between traits preferred by women for long-term relationships and men’s sociosexuality are scarce, do not specifically address the mentioned issue, and use a unidimensional approach to sociosexuality (Townsend, 1993; Sprecher et al., 2013; Szepsenwol et al., 2017). In this study, we tested whether economic resources, protection skills, and the willingness to allocate these resources in offspring—all traits preferred by women in long-term partners—are associated with men’s long-term mating orientation. Our results partially supported our predictions as we found that socioeconomic status was positively associated with a long-term mating orientation, but parenthood disposition did not moderate this effect. Instead, parenthood disposition seems to mediate this effect. Contrary to our predictions, socioeconomic status did not affect short-term mating orientation. Moreover, we failed to find the expected effects of strength on long-term mating orientation, but we found that strength was positively associated with short-term orientation.

Our first specific prediction was aimed at testing the effect of economic resources on long and short-term mating orientation and whether the willingness to allocate resources in offspring was moderating the effect for long-term oriented men. Regarding main effects, we found that resources, measured by socioeconomic status, were positively related to long-term mating orientation but did not affect short-term mating orientation. This result is aligned with previous studies that found the possession of resources attractive for women when choosing a long-term partner (Townsend, 1989; Buss et al., 1990; Sprecher et al., 1994; Buunk et al., 2002; Blossfeld and Timm, 2018) and support our prediction that resources are important in calibrating long-term mating orientation in men. In addition, our null result regarding short-term mating orientation is similar to the results of Sprecher et al. (2013), in which socioeconomic status was not related to short-term mating strategies but differs from the findings of Townsend (1993) that found a positive relationship. Our prediction regarding the positive relationship between economic resources and short-term mating orientation in men was based on previous evidence suggesting that economic resources may be a relevant trait for women when choosing a short-term mate (Greiling and Buss, 2000; Thomas and Stewart-Williams, 2018). But this preference for economic resources in short-term mating might be related to context-specific traits as when women meet a potential mate with higher socioeconomic status than their current partner (Greiling and Buss, 2000) or when the environment is high in resources (Thomas and Stewart-Williams, 2018). This circumstance may explain why, in a general context, economic resources seem to be related to long-term mating orientation in men but not to short-term mating orientation.

As important as resources are for women who seek long-term relationships, there are personality features that signal a willingness to allocate them to one partner and their offspring over a significant period (Buss, 2018; Webb and Fisher, 2018). In this regard, parenthood dispositions denote the willingness to invest in offspring and it is considered a component of men’s mate value which is especially relevant to attract partners for long-term relationships (Fisher et al., 2008). Accordingly, we expected that parenthood dispositions would moderate the effect of resources in the expression of long-term mating orientation in men. However, our results did not support this prediction, suggesting that socioeconomic status influences long-term mating orientation independently of parenthood dispositions. Since socioeconomic status and parenthood disposition were correlated in this study, we explored the possibility that parenthood disposition was mediating the relationship between socioeconomic status and long-term mating orientation. We found that indeed this was the case, suggesting that socioeconomic status affects long-term mating orientation through an increase in parenthood disposition. Future studies are needed to confirm that result due to its exploratory nature in this study.

Our second specific prediction was focused on testing the effect of protection skills on long and short-term mating orientation. In this regard, previous literature did not find an association between protection skills (i.e., strength and muscularity) and long-term sociosexual orientation, but it did for short-term (Lukaszewski et al., 2014; Polo et al., 2019). We proposed that this feature might have a positive effect on long-term mating orientation when interacting with other variables relevant for this context, like socioeconomic status and parenthood dispositions, and a direct and positive effect on short-term mating orientation. In line with previous research (Lukaszewski et al., 2014), strength predicted short-term but not long-term sociosexual orientation in men. However, contrary to our prediction, we did not find an effect for the proposed interaction between strength, socioeconomic status, and parenthood disposition. Thus, our results suggest that protection skills, measured from handgrip strength, do not seem to be a relevant factor that calibrates men’s long-term sociosexual orientation despite being described as relevant for women when choosing a long-term partner (Buss and Schmitt, 2019). A possible explanation is that, since it has been documented that protection skills are attractive for women in both mating contexts, men who display these traits may be attracting a larger pool of women and may gain more by pursuing a short-term mating strategy, maximizing the number of sexual partners (Hughes and Gallup, 2003; Frederick and Haselton, 2007; Lukaszewski et al., 2014; Polo et al., 2019). That can be particularly true in the case of men around their reproductive peak, as is the case of our sample, which was composed of young men with an average age of 22 years old. Finally, it is interesting to emphasize that strength is positively related to short-term mating orientation and has a null, but not negative, effect in the expression of long-term mating orientation. Strength has been associated with the possession of good genes and the ability to win a conflict (Sell et al., 2009), traits that are beneficial when pursuing a short-term mating strategy (Gangestad and Simpson, 2000). Consequently, it seems to be an important factor in calibrating short-term mating orientation but, according to our results, it did not affect long-term mating orientation. That suggests that a long-term mating orientation is not necessarily an alternative strategy employed when individuals cannot maximize their reproductive success through investing in casual sexual encounters. In addition, our results suggest that pursuing a short-term mating orientation by stronger men does not affect their orientation toward long-term mating and stress the importance of considering sociosexual orientation as a multidimensional construct.

To sum up, our results suggest that long-term-oriented men only display some of the traits that women prefer in them for long-term mating contexts, since resources but not protective skills are important for men’s long-term sociosexual orientation. Previous results showed that most of the traits preferred by women for short-term relationships are important in calibrating the expression of short-term mating orientation in men (Lukaszewski et al., 2014; Valentine et al., 2014; Polo et al., 2019). However, our results are less clear about the link between traits preferred for long-term mating and the expression of long-term mating orientation in men. This may indicate that, at least some individuals who possess traits reported to be attractive for long-term relationships, may be pursuing a short-term mating strategy instead. Possibly, this reflects the overall conflict of interests between mating strategies among the sexes that arise from differences in parental investment and potential reproductive rates (Trivers, 1972; Parker, 2006). Complementary theoretical approaches to the study of reproductive trade-offs in humans such as the life history theory (Kruger, 2017) may be useful to understand this variability in sociosexuality in future studies. From this framework, one of the variables that has been reported to affect sociosexual orientation in adulthood is the developmental conditions and, especially, the predictability and harshness of the childhood environment (Ellis et al., 2009). Considering these developmental conditions jointly with current traits and conditions may help to have a wider understanding of the causes of the individual differences in mating strategies.

Our study has several limitations. First, our measure of parenthood dispositions which, although based on a subscale of a validated questionnaire (Fisher et al., 2008), might be too general as it was composed of only two items and precluded delving into different sources of investment and commitment to offspring. Future studies should include more specific measures of parenthood dispositions to determine whether different types of investment in offspring influence the relationships between socioeconomic status and long-term mating orientation in different ways. Second, our sample mainly consisted of young men and with low variability in age. This precludes analyzing whether the association of traits and mating strategies changes as individuals age and consolidate their social status. Future studies should include a wider age range to address this issue, either by pursuing a larger sample or by quota sampling by age groups. Our third limitation is that we did not have information about whether the participants currently had children or not. Despite that our sample was composed mainly of young men, their paternal status may be relevant as there is some evidence suggesting that unrestricted sociosexuality is reduced during parenthood in men, but only in those that reside with their children (Gettler et al., 2019).

## Conclusion

In conclusion, our study found that resources are a relevant trait related to the expression of long-term sociosexual orientation in young men but not for the expression of short-term sociosexual orientation. The effect of resources over long-term mating orientation is probably mediated but not moderated by parenthood dispositions. In contrast, protection skills are important traits only concerning short-term sociosexual orientation. Finally, this study not only provides evidence of the features exhibited by men regarding long-term mating orientation in a Latin American context but also stresses the importance of considering sociosexual orientation as a multidimensional construct to better understand the complexity of human mating.

## Data Availability Statement

The original contributions presented in the study are included in the article/Supplementary Material, further inquiries can be directed to the corresponding author/s.

## Ethics Statement

The studies involving human participants were reviewed and approved by Comité Ético Científico from Universidad de Playa Ancha. The patients/participants provided their written informed consent to participate in this study.

## Author Contributions

GF, PP, and JM-R conceived and designed the study. PP and JM-R collected the data. GF performed the statistical analysis. GF and PP wrote the first draft of the manuscript. All authors contributed to manuscript revision and edit some parts of it, read, and approved the submitted version.

## Conflict of Interest

The authors declare that the research was conducted in the absence of any commercial or financial relationships that could be construed as a potential conflict of interest.

## Publisher’s Note

All claims expressed in this article are solely those of the authors and do not necessarily represent those of their affiliated organizations, or those of the publisher, the editors and the reviewers. Any product that may be evaluated in this article, or claim that may be made by its manufacturer, is not guaranteed or endorsed by the publisher.
