# Supplementary material for: Long-Term Mating Orientation in Men: The Role of Socioeconomic Status, Protection Skills, and Parenthood Disposition
Source: Front Psychol. 2022 Feb 25;13:815819. doi: 10.3389/fpsyg.2022.815819 (PMC8913582; doi:10.3389/fpsyg.2022.815819)
Supplement: Supplementary file 2 [file Data_Sheet_2.DOCX]

The database can be found at: https://osf.io/42sf8/?view_only=4d5b0487912f4bc49ce942c503ae098b
