# Supplementary material for: Long-Term Mating Orientation in Men: The Role of Socioeconomic Status, Protection Skills, and Parenthood Disposition
Source: Front Psychol. 2022 Feb 25;13:815819. doi: 10.3389/fpsyg.2022.815819 (PMC8913582; doi:10.3389/fpsyg.2022.815819)
Supplement: Supplementary file 3 [file Table_1.docx]

Supplementary table: Complete generalized linear models for long and short-term sociosexual orientation (N = 197).

|  | **FIRST HYPOTHESIS: TESTING SOCIOECONOMIC STATUS** | | | | | | | |
| --- | --- | --- | --- | --- | --- | --- | --- | --- |
|  |  | |  | | **Long-Term Orientation** | | **Short-Term Orientation** | |
| **Main effects** | **Global status** | | **Coeff. estimate**  **Standard error**  **t-value**  **Pr > \|t\|** | | 1.38  0.43  3.24  0.001 | | 0.46  0.61  0.76  0.451 | |
|  | **Age** | | **Coeff. estimate**  **Standard error**  **t-value**  **Pr > \|t\|** | | 0.01  0.16  0.07  0.942 | | 0.29  0.23  1.28  0.202 | |
|  | **In a relationship** | | **Coeff. estimate**  **Standard error**  **t-value**  **Pr > \|t\|** | | 0.55  1.45  0.38  0.704 | | -6.28  2.07  -3.03  0.003 | |
|  | **Intercept** | | **Coeff. estimate**  **Standard error**  **t-value**  **Pr > \|t\|** | | 30.71  4.39  6.99  0.000 | | 37.87  6.27  6.04  0.000 | |
|  | **AIC** | | | | 1462.8 | | 1602.8 | |
| **Interaction effects** | **Global status** | | **Coeff. estimate**  **Standard error**  **t-value**  **Pr > \|t\|** | | 1.23  1.64  0.75  0.453 | | -- | |
|  | **Parenthood disposition** | | **Coeff. estimate**  **Standard error**  **t-value**  **Pr > \|t\|** | | 1.55  0.85  1.84  0.068 | | -- | |
|  | **Age** | | **Coeff. estimate**  **Standard error**  **t-value**  **Pr > \|t\|** | | 0.02  0.15  0.18  0.859 | | -- | |
|  | **In a relationship** | | **Coeff. estimate**  **Standard error**  **t-value**  **Pr > \|t\|** | | 0.73  1.37  0.54  0.593 | | -- | |
|  | **Global status * Parenthood disposition** | | **Coeff. estimate**  **Standard error**  **t-value**  **Pr > \|t\|** | | -0.04  0.15  -0.286  0.775 | | -- | |
|  | **Intercept** | | **Coeff. estimate**  **Standard error**  **t-value**  **Pr > \|t\|** | | 16.93  9.89  1.71  0.089 | | -- | |
|  | **AIC** | | | | 1439.8 | | -- | |
|  | **SECOND HYPOTHESIS: TESTING PROTECTION SKILLS (STRENGTH)** | | | | | | | |
|  |  |  | | **Long-Term Orientation** | | **Short-Term Orientation** | | |
| **Main effects** | **Strength** | **Coeff. estimate**  **Standard error**  **t-value**  **Pr > \|t\|** | | -0.06  0.10  -0.57  0.569 | | 0.28  0.13  2.06  0.041 | | |
|  | **BMI** | **Coeff. estimate**  **Standard error**  **t-value**  **Pr > \|t\|** | | -0.09  0.23  -0.41  0.679 | | -0.26  (0.32)  -0.81  0.419 | | |
|  | **Age** | **Coeff. estimate**  **Standard error**  **t-value**  **Pr > \|t\|** | | 0.04  0.18  0.24  0.813 | | 0.36  0.25  1.44  0.151 | | |
|  | **In a relationship** | **Coeff. estimate**  **Standard error**  **t-value**  **Pr > \|t\|** | | 0.05  1.49  0.03  0.973 | | -6.62  2.05  -3.23  0.001 | | |
|  | **Intercept** | **Coeff. estimate**  **Standard error**  **t-value**  **Pr > \|t\|** | | 42.95  6.26  6.86  0.000 | | 33.49  8.63  3.88  0.000 | | |
|  | **AIC** | | | 1474.6 | | 1600.8 | | |
| **Interaction effects** | **Global status** | **Coeff. estimate**  **Standard error**  **t-value**  **Pr > \|t\|** | | 4.47  10.42  0.43  0.669 | | -- | |  |
|  | **Parenthood disposition** | **Coeff. estimate**  **Standard error**  **t-value**  **Pr > \|t\|** | | -0.69  5.26  -0.13  0.896 | | -- | |  |
|  | **Strength** | **Coeff. estimate**  **Standard error**  **t-value**  **Pr > \|t\|** | | -0.05  1.26  -0.04  0.971 | | -- | |  |
|  | **BMI** | **Coeff. estimate**  **Standard error**  **t-value**  **Pr > \|t\|** | | -0.19  0.21  -0.89  0.375 | | -- | |  |
|  | **Age** | **Coeff. estimate**  **Standard error**  **t-value**  **Pr > \|t\|** | | 0.06  0.17  0.38  0.705 | | -- | |  |
|  | **In a relationship** | **Coeff. estimate**  **Standard error**  **t-value**  **Pr > \|t\|** | | 0.55  1.38  0.40  0.691 | | -- | |  |
|  | **Global status * Parenthood disposition** | **Coeff. estimate**  **Standard error**  **t-value**  **Pr > \|t\|** | | 0.11  0.94  0.12  0.906 | | -- | |  |
|  | **Global status * Strength** | **Coeff. estimate**  **Standard error**  **t-value**  **Pr > \|t\|** | | -0.06  0.23  -0.26  0.799 | | -- | |  |
|  | **Parenthood disposition * Strength** | **Coeff. estimate**  **Standard error**  **t-value**  **Pr > \|t\|** | | 0.06  0.12  0.51  0.614 | | -- | |  |
|  | **Global status * Parenthood disposition * Strength** | **Coeff. estimate**  **Standard error**  **t-value**  **Pr > \|t\|** | | -0.005  0.02  -0.24  0.811 | | -- | |  |
|  | **Intercept** | **Coeff. estimate**  **Standard error**  **t-value**  **Pr > \|t\|** | | 18.97  56.35  0.34  0.737 | | -- | |  |
|  | **AIC** | | | 1443.8 | | -- | |  |
